# Germline factors, TDRD and Piwi, colocalize with Vasa on the mitotic apparatus during the embryogenesis of the sea urchin

**DOI:** 10.1016/j.ydbio.2025.07.016

**Published:** 2025-07-29

**Authors:** Mariana Witmer, Nirali Mehta, Natsuko Emura, Mamiko Yajima

**Affiliations:** Department of Molecular Biology Cell Biology Biochemistry, Brown University, 185 Meeting Street, BOX-GL277, Providence, RI, 02912, USA

## Abstract

Germline factors are thought to function exclusively in the germline, providing the unique characteristics of germ cells. However, recent studies suggest that some of these factors may also be expressed and function outside the germline. One such example includes Vasa, a DEAD-box RNA helicase that appears to control localized translation on the spindle, facilitating efficient protein synthesis during embryogenesis of the sea urchin. However, it remains unclear if other germline factors are also involved in this process. In this study, we investigated the localization dynamics of Vasa’s partners in the germline, such as Tudor-domain-containing proteins (TDRDs) and P-element-induced wimpy testis proteins (Piwis). Among TDRDs tested in this study, we found that TDRD7 is enriched on the spindle and forms granules with Vasa during early embryogenesis. Vasa and TDRD7 recruited each other when the expression of either was forced at the membrane, suggesting their interaction with each other. TDRD7 mutants lacking the N-terminal eLOTUS domain or the central intrinsically disordered region exhibited reduced granule formation, which also compromised their recruitment to Vasa. In contrast, PiwiL1/2 and PiwiL3 showed enrichment at the perinuclear region and the spindle, yet were never recruited to Vasa or TDRD7 when either was expressed at the membrane. These results suggest that a group of germline factors is present and may dynamically interact with each other on the spindle, contributing to somatic cell regulation in the sea urchin embryo.

## Graphical Abstract

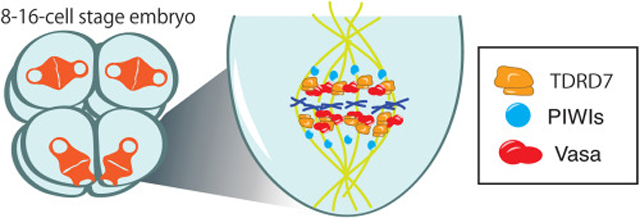

## 1. Introduction

Germ cells of most animals contain membraneless compartments called germ granules that consist of multiple proteins and RNA necessary for the function and development of germ cells (Voronina et al., 2011; Gao and Arkov, 2013; Dodson and Kennedy, 2020). The known roles of germ granules include transposon silencing, mRNA surveillance and regulation, mRNA localization, and mRNA storage. The formation and localization of these granules are thought to allow for these processes to occur at the optimal space and time in the germ cell (Voronina et al., 2011; Gao and Arkov, 2013; Dodson and Kennedy, 2020). In *Drosophila,* it has been found that germ granules contain various germline factors, such as Vasa, several Tudor-domain containing proteins (TDRDs), and P-element-induced wimpy testis proteins (Piwis) (Harris and Macdonald, 2001; Voronina et al., 2011). These proteins are reported to be involved in PIWI-interacting RNA (piRNA) biogenesis, including the piRNA ping-pong pathway in the granules, which subsequently contributes to the piRNA-mediated transposon and mRNA silencing mechanism in the germline (So et al., 2021; Vourekas et al., 2016).

The TDRD proteins have regions identified as LOTUS domains that are highly conserved among eukaryotes and bacteria. In *Drosophila*, these include Oskar, Tejas (TDRD5 ortholog), Tapas (TDRD7 ortholog), and meiosis regulator and mRNA stability factor 1 (MARF1) (Jeske et al., 2017; Kubíková et al., 2020). Two different types of LOTUS domains have been proposed: extended LOTUS (eLOTUS) and minimal LOTUS (mLOTUS) domains. mLOTUS domains are responsible for RNA interaction and have been shown to bind G-rich RNAs *in vitro*, while eLOTUS domains have been shown to play a role in protein-protein interactions with Vasa (Jeske et al., 2015, 2017; Kubíková et al., 2020). Previous reports suggest that eLOTUS domains have an additional C-terminal alpha helix, allowing their interaction with Vasa (Jeske et al., 2017; Ding et al., 2020). Therefore, it has been proposed that Oskar recruits Vasa into the pole granule through its eLOTUS domain, yet the mechanistic detail is yet to be known (Jeske et al., 2017).

The expression of these germline factors is often most prominent in the germline, and it has been considered to be involved in the biological processes unique to the germline (Lasko, 2013; Parvinen, 2005; Castrillon et al., 2000; Jeske et al., 2017). However, growing evidence in various models suggests that they may also be expressed and function outside of the germline under certain circumstances, including during regeneration, embryonic regulation, and some cancer cell regulation (Ross et al., 2014; Rojas-Rios and Simonelig, 2018; Sato and Siomi, 2020; Poon et al., 2016). These biological events are connected to the regulation of multipotent cells, and therefore, the expression of germline factors is thought to play a role in this process. However, the functional details of these germline factors and whether they work together as they do in the germline during somatic cell regulation remain poorly understood.

In this study, we use sea urchin embryos as an ideal model to start addressing these remaining questions in the field. In the sea urchin embryo, we previously reported that Vasa, one of the germline factors conserved across organisms, is expressed and functions in localized mRNA translation on the spindle during embryogenesis (Fernandez-Nicolas et al., 2022). In this process, Vasa is expressed in both somatic and primordial germ cell (PGC) lineages, and its knockdown inhibits overall translation and cell cycle progression, thereby affecting the entire embryonic development beyond the germline (Fernandez-Nicolas et al., 2022). Furthermore, the ectopic localization of Vasa at the membrane promotes ectopic protein synthesis onsite, which then causes developmental failure. Therefore, we hypothesize that Vasa plays a critical role in regulating protein synthesis specifically on the spindle, contributing to precise control of protein production necessary for dynamic embryonic cell regulation. However, we still do not know whether Vasa acts alone in this process for the somatic function or whether other germline factors are also involved.

To address this question, this study focuses on two germline factor families, Piwis and TDRDs, which are known to interact with Vasa in the *Drosophila* germline. We examined the localization dynamics of these germline factors and their potential interactions with Vasa on the spindle during somatic cell development of the sea urchin embryo. We found that Vasa and TDRD7 form granules on the spindle and recruit each other even at ectopic sites, such as the membrane. Piwis showed similar enrichment on the spindle but with less granule formation or interaction with Vasa. These observations suggest that a group of germline factors is present on the mitotic spindle, potentially contributing together to the regulation of multipotent somatic cells during embryogenesis.

## 2. Materials and methods

### 2.1. Animals and embryo culture

The purple sea urchin, *Strongylocentrotus purpuratus (S. purpuratus*) was obtained through vendors in California (Pete Halmey and Marinus Scientific, LLC). Embryos were obtained by injecting 1 mL of 0.5 M KCl to induce gamete spawning, in which eggs and sperm were obtained from a single male and female. Eggs were fertilized in 1 mM 3-aminotriazole (Millipore-Sigma, St. Louis, MO, USA) to remove fertilization envelopes. A single layer of fertilized eggs was cultured in 16 °C filtered seawater in Petri dishes until the desired developmental stage.

### 2.2. Blast and motif analysis

The protein sequences of TDRD1, TDRD7, TDRD9, TDRD12, PIWIL1, PIWIL2, and PIWIL3 of the sea urchin, *S. purpuratus*, were obtained from echinobase.org (Telmer et al., 2024). TDRD5 and TDRD7 of the human (*H. sapiens*), mouse (*M. musculus*), frog (*X. tropicalis*), and fruit fly (*D. melanogaster*) as well as Vasa of the human (*H. sapiens*), mouse (*M. musculus)*, silkworm (*B. mori*), and fruit fly (*D. melanogaster*) sequences were obtained from NCBI. Protein sequence alignments and molecular phylogenetic trees were constructed using Clustal Omega. Protein structural motif analysis was performed through the NCBI blast search using the default settings. For further details, please also see Table S1.

### 2.3. Cloning and plasmid construction

The open reading frame (ORF) of TDRD1, TDRD7, TDRD9, TDRD12, PIWIL1/2, and PIWIL3 was cloned using the cDNA library prepared from eggs or 16-cell embryos of *S. purpuratus*. The High fidelity taq (HiFi PCR premix, Clontech # 639298) was used for the PCR reaction. The amplified PCR products were then cloned into the TOPO (topoisomerase-based cloning) vector using the Zero Blunt TOPO PCR cloning kit (Fisher Scientific #45–124-5). The resulting clones were sequenced and validated for the correct sequence for each gene of interest. Each ORF was then subcloned into the SP64-GFP vector to generate SP64-GFP-TDRD1, -TDRD7, -TDRD9, -TDRD12, -PIWIL1/2, and -PIWIL3 using InFusion Snap Assembly (Clontech # 638945) by following the manufacturer’s protocol.

To create GFP-TDRD7 mutant constructs, each TDRD7 mutant fragment was amplified by PCR (HiFi PCR premix, Clontech # 639298) from the TDRD7 full-length construct or synthesized (Integrated Device Technology, IDT), using primers or gBlocks, respectively, listed in Table S2. Each TDRD7-mutant fragment was then inserted at the 3’end of GFP ORF into the SP64-GFP vector, using InFusion Snap Assembly (Clontech # 638945). Membrane-mCherry TDRD7 full length and mutants were made by inserting each TDRD7 fragment at the 3’end of mCherry ORF into the SP64-membrane-mCherry vector (Fernandez-Nicolas et al., 2022), using InFusion Snap Assembly (Clonetech # 638945). Plasmids were sequenced, and positive clones were used for the experiments. All Vasa-related constructs or 2-xmCherry-EMTB were previously made (Fernandez-Nicolas et al., 2022) or obtained through Addgene (#2674; von Dassow et al., 2009). For further details, please also see Table S2.

### 2.4. In vitro transcription, microinjection, and microscopy

Plasmids were linearized with *Xba*I, *Bam*HI, or *Sal*I and transcribed *in vitro* with SP6 mMessage mMachine kit (#AM1340, Invitrogen, USA). Fertilized eggs were injected with 100–1000 ng/μL stock of mRNA for GFP-SpTDRD1, TDRD7, TDRD9, TDRD12, PIWIL1/2, and PIWIL3, SpVasa-mCherry, SpVasa-GFP, or 2xmCherry-EMTB (Addgene #26742). Please see the figure legends for the exact dose of mRNA used for each experiment. Please also see Table S3 for additional details. Injected embryos were incubated in filtered seawater at 16 °C until they reached the desired stage of development. Live imaging was taken under the Nikon CSU-W1 Spinning disk laser microscope. Each sample group was imaged using a 40x or 60x objective within 30 min to ensure consistent developmental timing and cell cycle stage across sample groups and replicates. Additionally, each image was typically captured with 30 z-stacks at 1 μm intervals, covering the entire blastomere facing the objective. Each imaging cycle generally produced 15–30 embryo images per sample group. Every imaging experiment was performed at least three times.

### 2.5. Antibody construction and immunoblot

Custom-made affinity-purified rabbit antibodies against TDRD7 and TDRD5 were made by Genscript (Piscataway, NJ). The peptide antigen sequence for SpTDRD7 (102–116 amino acids) is underlined in Fig. S1B, and SpTDRD5 (458–471 amino acids) is underlined in Fig. S2A. Affinity-purified rabbit polyclonal Human PiwiL4 unconjugated antibodies and mouse clonal β-actin antibodies were purchased from Thermo Scientific (Catalog #PA5–31448) and Cell Signaling Technology (#8H10D10), respectively. Five microliters of lysate from eggs and 16-cell stage embryos were run on a 10 % polyacrylamide gel and transferred onto a nitrocellulose membrane, which was then blocked with 5 % Bovine Serum Albumin (BSA). The membranes were incubated overnight at 4 °C with primary antibodies TDRD7, PiwiL4 and TDRD5 at dilutions of 1:500 or 1:1000, respectively, and with actin antibody at a 1:5000 dilution in 1.2 % BSA, or with 0 % BSA for controls. Afterward, the membranes were washed five times with Tris-buffered saline containing 0.1 % Tween^®^ 20 (TBST). They were then incubated with HRP-conjugated anti-rabbit (Cell Signaling Technology, #7047) for TDRD7 and TDRD5, or with anti-mouse secondary antibody (Cell Signaling Technology, #7076) for β-actin. The proteins were detected using a chemiluminescence substrate (SuperSignal^™^ West Pico PLUS Chemiluminescent Substrate, ThermoFisher, Cat #34577) and imaged with a ChemiDoc Gel Imaging System (BioRad, USA).

### 2.6. Immunofluorescence

Embryos were fixed with 90 % methanol for overnight at −20 °C, rinsed with 1X PBS, and incubated in the primary antibody overnight at 4 °C, followed by 10 washes with 1X PBS, then incubated with the secondary antibody at 4 °C overnight. Primary antibodies were used at a dilution of 1:100 anti-PIWI (Thermo Fisher Scientific #PA5–31448) with 1 % BSA, 1:100 anti-TDRD7, and 1:300 β-Tubulin (Sigma-Aldrich, #F2043). The secondary antibody was used at a dilution of 1:300 Alexa 488-conjugated goat anti-rabbit or -mouse, 1:300 Alexa 555-conjugated goat anti-rabbit or -mouse, Cy3 goat anti-rabbit or -mouse, and 1:300 FITC-β-Tubulin (Sigma-Aldrich, #F2043). The secondary antibody was then washed 8 times with 1X PBS, and Hoechst (Thermo Fisher Scientific # 62249) treatment to stain DNA was performed at a dilution of 1:1000 for 15 min. Samples were plated onto slides. All fluorescent images were taken under the Nikon CSU-W1 Spinning disk laser microscope.

### 2.7. Quantitative RT-PCR

Embryos were collected at the desired developmental stage and were subjected to total RNA extraction with RNeasy Plus Mini Kit(Qiagen, #74034), followed by cDNA library preparation using 5X PrimeScript (Takara, #RR036A). cDNA was then used for quantitative PCR (qPCR) using Luna Universal qPCR Master mix (New England BioLabs, #M3003X). Primers designed in the coding region of respective TDRDs are listed in Table S2.

### 2.8. AlphaFold predictions

All structure protein sequences were obtained through echinobase.org (Telmer et al., 2024). The structure was predicted using *AlphaFold 3* software (https://alphafoldserver.com/). Structures were then visualized using *PyMol*.

### 2.9. Image analysis

All image analyses were performed using either *Image J* or Nikon NIS Elements. Colocalization analysis was performed using NIS Elements Software. To minimize technical variations, embryo images with similar angles and cell cycle stages were pre-selected and used for the downstream analysis. Ranges of the z-planes (20–30 stacks with 1 μm intervals) encompassing the spindle of M-phase embryos, the perinuclear regions of S-phase embryos, or the whole blastomere of the embryos were analyzed. Binary layers for each laser channel were selected through thresholding to reduce the background and select the signal intensities correlating to granule structures. Colocalization intensity analysis was performed by identifying the colocalized areas through binary layer thresholding for each channel, followed by measuring the signal intensity within the colocalized areas.

### 2.10. Statistical analysis

Statistical significance was performed by PRISM (GraphPad) using a *t*-test or one-way or two-way ANOVA. *P* values less than 0.05 were considered significant. Asterisks were used to indicate significance against the control group located in the leftmost column for all experiments, corresponding with * is *P* < 0.05, ** is *P* < 0.01, *** is *P* < 0.001, **** is *P* < 0.0001. For bar graphs, each dot represents data from a single embryo unless indicated otherwise in the figure legend.

## 3. Results

### 3.1. Characterization of TDRD proteins in purple sea urchin embryos

Humans have at least 36 proteins that contain Tudor domains (Qin et al., 2013). In the purple sea urchin (*Strongylocentrotus purpuratus*), based on the database (echinobase.org; Telmer et al., 2024), 15 proteins contain Tudor domains. Among those, eight are annotated as TDRDs, including TDRD1 (LOC#100887863), TDRD3 (LOC#585351), TDRD4 (LOC#593101), TDRD5 (LOC#578673), TDRD6 (LOC#575181), TDRD7 (LOC#577641), TDRD9 (LOC#100891933), TDRD12 (LOC#105444112). The phylogram analysis of sea urchin (Sp) and human (Hs) TDRDs suggests that each SpTDRD is most closely related to its human ortholog, except for SpTDRD6, which is instead more closely related to HsTDRD1 (Fig. S1A).

It is proposed that the eLOTUS domain is critical for TDRD’s interaction with Vasa in *Drosophila*. In *Drosophila*, Tejas/TDRD5, Tapas/TDRD7, and Oskar have the eLOTUS domain (Jeske et al., 2017). While we found no protein sequence in *S. purpuratus* similar to Oskar, the protein sequence analysis of SpTDRD7 and HsTDRD7 suggests that both proteins are relatively conserved in sequence for their functional domains, such as the eLOTUS and Tudor domains (Fig. S1B). The NCBI protein blast search predicts that HsTDRD7 has one eLOTUS domain, two mLOTUS domains, and three Tudor domains (Fig. S1C). In contrast, SpTDRD7 has only one eLOUTS domain and one Tudor domain, making its sequence shorter (677 aa) than that of HsTDRD7 (1098 aa).

Further, the *AlphaFold 3* prediction suggests that the first LOTUS domain of SpTDRD7 contains the fourth alpha helix (Fig. S1D–E), which is a unique characteristic of the eLOTUS domain (Jeske et al., 2017). However, this domain was not predicted in the HsTDRD7 structure, though it is present in the fly (Dm) TDRD7 structure (Fig. S1E). Furthermore, *IUPRED3* predicts that a long intrinsically disordered region (IDR) exists in the middle region from 97 aa to 283 aa, and a short IDR is found between 332 aa and 360 aa of SpTDRD7 (Fig. S1C). Similarly, SpTDRD5 also shows mild sequence similarity to HsTDRD5 (Fig. S2A). In this case, however, SpTDRD5 appears to have an additional mLOTUS domain compared to HsTDRD5 (Fig. S2B). Within the first LOTUS domain, SpTDRD5 seems to include the fourth alpha helix, suggesting it may also possess the eLOTUS domain (Fig. S2C–D). Multiple predicted IDR regions also seem to be present between the structured domains from 117 aa to 194 aa, 268 aa to 378 aa, and 794 aa to 900 aa, as well as a large IDR at the C-terminus from 1147 aa to 1459 aa of SpTDRD5 (Fig. S2B).

### 3.2. TDRD5 and TDRD7 form granules on the spindle during embryogenesis of the sea urchin

**Fig. 1.**
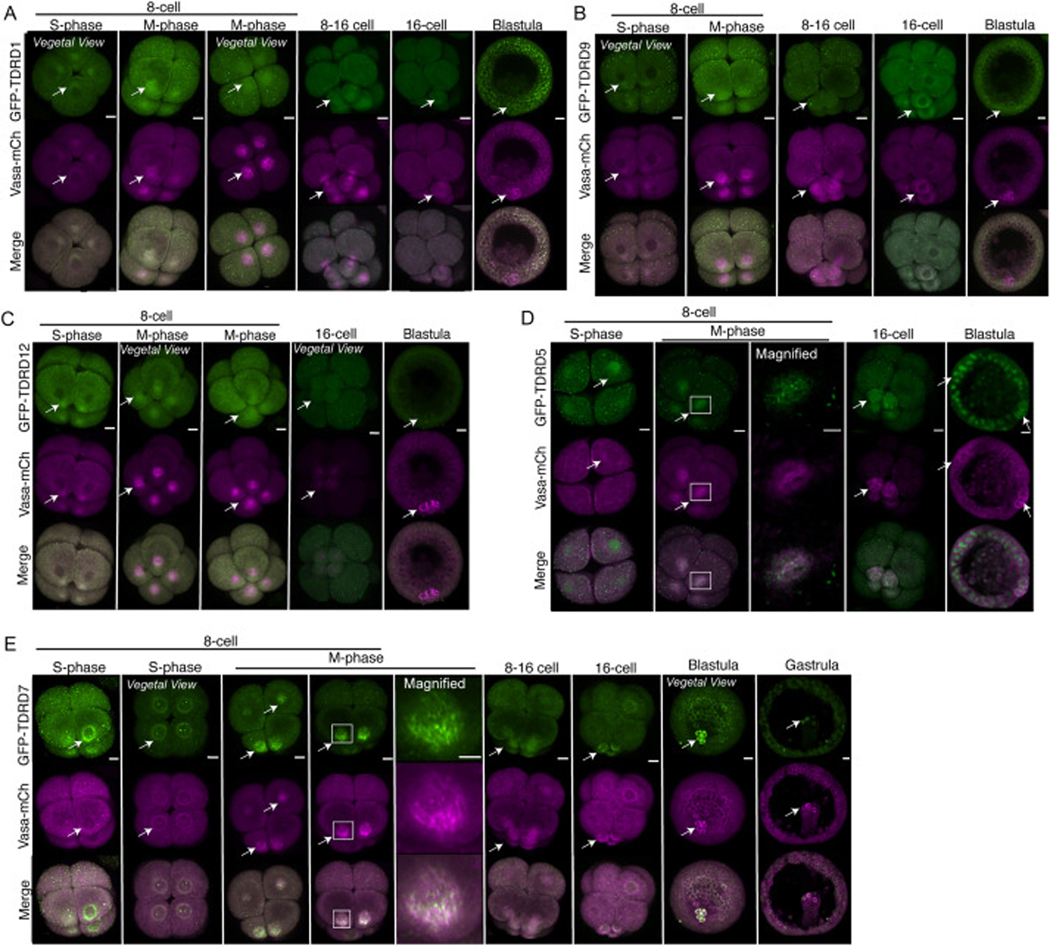
Localization of S. purpuratus TDRD1, TDRD5, TDRD7, TDRD9, and TDRD12. Confocal images of GFP-tagged TDRDs and Vasa-mCherry in the 8-cell to the blastula stage embryos. 500ng/μL stock of each mRNA was microinjected into zygotes. (A) GFP-TDRD1 localization. Images obtained over three replicate, independent rounds. n = 40 (8–16 cell), n = 36 (blastula). 85 % of S-phase embryos, 76 % of M-phase embryos, and 100 % of blastula embryos exhibited a similar localization signal as shown in the image. (B) GFP-TDRD9 localization. n = 38 (8–16 cell), n = 25 (blastula). 100 % of S-phase embryos, 81 % of M-phase embryos, and 100 % of blastula and gastrula embryos exhibited a similar localization signal as demonstrated in the image. (C) GFP-TDRD12 localization. n = 33 (8–16 cell), n = 18 (blastula). 55.55 % of S-phase embryos, 66.67 % of M-phase embryos, and 88 % of blastula embryos exhibited a similar localization signal as shown in the image. (D) GFP-TDRD5 localization. n = 39 (8–16 cell), n= 27 (blastula). 60 % of S-phase embryos,83 % of M-phase embryos, and 100% of the blastula embryos exhibited a similar localization signal as shown in the image. (E) GFP-TDRD7 localization. n = 37 (8–16 cell), n = 24 (blastula). 100 % of S-phase, M-phase, and blastula embryos exhibited a similar localization signal as shown in the image. All experiments were conducted in at least three independent cycles. All scale bars in this figure = 10 μm.

To visualize the SpTDRD localization dynamics, we cloned the open reading frame (ORF) of each SpTDRD (hereafter referred to as TDRD). Among eight TDRDs in the database, we cloned TDRD1, TDRD5, TDRD7, TDRD9, and TDRD12, which are reported to be involved in piRNA biogenesis (Chuma et al., 2006; Shoji et al., 2009) and germline development in *Drosophila* and mice (Ishizu et al., 2012; Mathioudakis et al., 2012; Siomi et al., 2010)and fused each ORF to GFP. We microinjected mRNAs of each of GFP-TDRDs and Vasa-mCherry into sea urchin zygotes imaged at the 8–16-cell (4–4.5 h post fertilization; hpf), and blastula (24 hpf) stages. The resultant images show that TDRD1, TDRD9, and TDRD12 localize perinuclearly during S-phase at the 8-cell stage and are slightly enriched at the vegetal pole during the 8–16-cell stage M-phase of all blastomeres, and then in micromeres that are formed through an asymmetric cell division at the 16-cell stage (Fig. 1Figure figs1A–C, arrows). Micromeres undergo another asymmetric cell division at the 32-cell stage, hence contributing to the PGCs (Voronina et al., 2008), while the rest of the embryonic cells contribute to the somatic lineages. Therefore, TDRDs appear to be localized on the spindle of somatic cells and the PGCs. These expression dynamics are similar to those of Vasa. However, these TDRD proteins appeared less granular than Vasa at these stages. At the blastula stage, TDRD1 appeared perinuclear and granular throughout the embryo, unlike Vasa, which is more enriched in the germline at this time point of development (Fig. 1A, arrow). TDRD9 and TDRD12 showed no specific localization at the blastula stage (Fig. 1B–C).

In contrast, TDRD5 and TDRD7 displayed granular signals on the spindle and at the perinuclear region of all blastomeres during the 8–16-cell stage (Fig. 1D–E, arrows). TDRD5 exhibited a granule signal during M-phase, albeit less distinctly than TDRD7 and Vasa. Further, it was predominantly localized in the nucleus during the S-phase throughout development (Fig. 1D, arrows) and was slightly enriched in the micromere at the 16-cell stage. Notably, the signal for GFP-TDRD5 was extremely dim throughout development. Yet, the increased dose (up to 1200 ng/μL stock) of GFP-TDRD5 mRNA introduction resulted in abnormal development and was lethal at Day 1. Therefore, the endogenous TDRD5 protein may be little expressed during early embryogenesis of the sea urchin. Overall, among TDRDs tested in this study, TDRD7 showed the clearest enrichment in micromeres at the 16-cell stage and in the germline at the blastula and gastrula (48 hpf) stages (Fig. 1E, arrows).

Since no commercial antibody that cross-reacts with any of SpTDRDs is available, we made peptide antibodies against SpTDRD5 and SpTDRD7 using the unique antigen region of each protein (Figs. S1 and S2, underlines). The SpTDRD7 antibody showed a specific band at the expected size of 76 kDa by immunoblot (Fig. S3A). The immunofluorescence showed the signal localization on the spindle, at perinuclear, and in the germline, which is similar to the localization dynamics of GFP-TDRD7 (Fig. S3B). In contrast, the SpTDRD5 antibody showed no detectable signal by immunoblot (data not shown). Immunofluorescence of this antibody shows a slight signal in the nucleus, which is consistent with GFP-TDRD5 imaging results (Fig. S3C).

The expression profiles of the *tdrd* mRNA transcripts were available except for *tdrd7* in the database (echinobase.org), which shows similar patterns across *tdrds*, starting higher in eggs (0 hpf) and declining toward the blastula stage (24 hpf) (Fig. S3D). This trend appears to be consistent with our RT-qPCR results (Fig. S3E). In contrast, the database suggests that *tdrd5* transcript levels are higher than those of other *tdrds*, while our RT-qPCR data indicate that all *tdrds* have similar expression levels in eggs and 16-cell stage embryos. However, only *tdrd7* shows an increased level at the blastula stage (Fig. S3F). These observations suggest that, except for the elevated *tdrd7* level at the blastula stage, all *tdrd* transcripts tested in this study have comparable expression levels during early embryogenesis. Therefore, the low detection of TDRD5 protein may be independent of its transcript levels. Transcript and protein levels do not always align during early embryogenesis due to significant maternal contributions. Hence, active degradation or translation inhibition of TDRD5 may be present in the early embryo. Alternatively, the TDRD5 antibody used in this study might have low immunogenicity, although this does not explain the low expression levels of exogenous GFP-TDRD5 protein. Further investigation is needed to clarify all of these possibilities in the future. Overall, TDRD7 appears to exhibit the most prominent expression and similar localization dynamics to Vasa among the TDRDs tested in this study. Therefore, we focused on TDRD7 for the remainder of this study.

### 3.3. Vasa localizes with TDRD7 on the spindle and interacts with each other

**Fig. 2.**
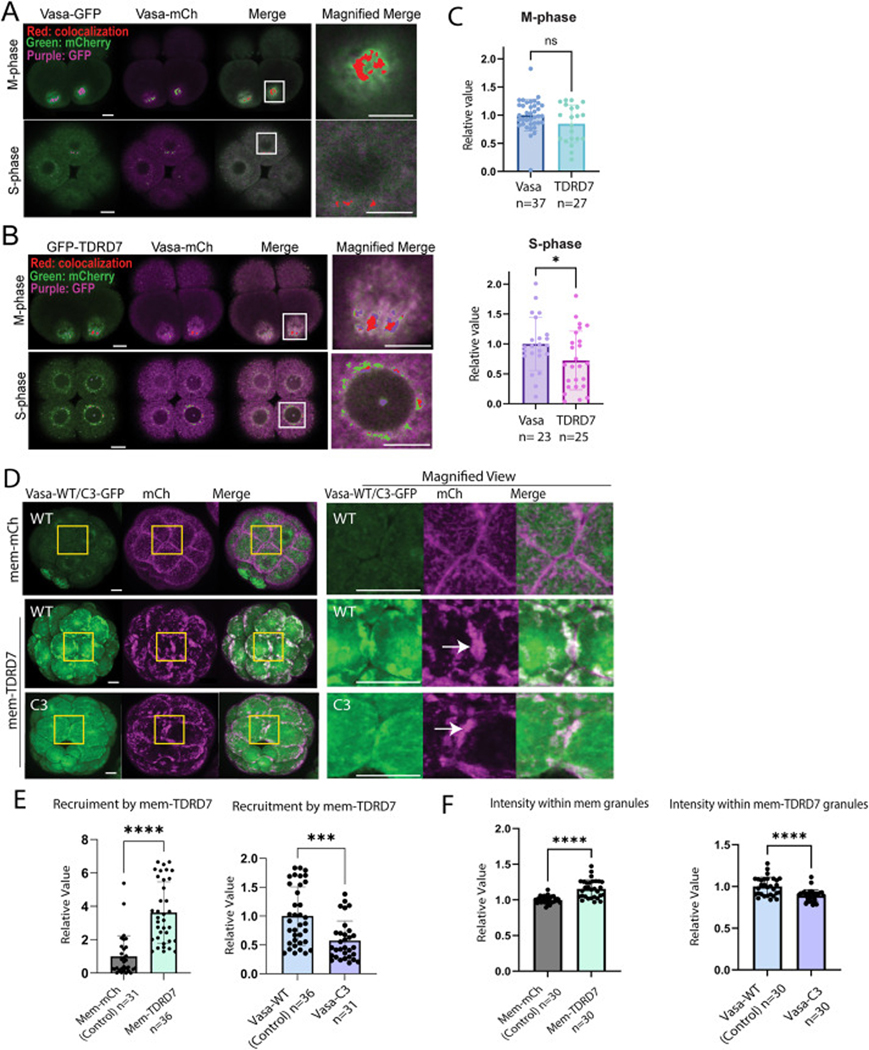
TDRD7 colocalizes with Vasa on the spindle and recruits Vasa through the C3 region. (A–B) Representative images of colocalization analysis for Vasa-GFP and Vasa-mCherry (mCh) (A) and GFP-TDRD7 and Vasa-mCh (B) during the M- and S-phases at 8–16 cell stage embryos. 500ng/μL stock of each mRNA was microinjected into zygotes. Granules above the threshold are shown in green for the mCh channel, purple for the GFP channel, and red for the colocalized signals between the green and purple signals. (C) Quantitative analysis of the granule colocalization between Vasa-mCh and Vasa-GFP (control) or GFP-TDRD7 granules at the M (top) and S (bottom) phases. Each value was normalized to the mean value of the control group. (D) Representative images of Vasa-WT/C3 (green; 500 ng/μL stock) recruitment by mem-mCh or mem-TDRD7 (magenta; 1000 ng/μL stock). Squared regions are shown in the magnified views on the right. Arrows indicate a region with recruitment. (E–F) Colocalization (E) and colocalization intensity analysis (F) of mem-mCh/TDRD7 and recruited Vasa-GFP and of mem-TDRD7 and recruited Vasa-WT/C3. Each value was normalized to the mean value of the control group in each graph. An unpaired *t*-test was used for statistical analysis for all graphs. The dots in each column of the graph indicate individual embryos. n in the graphs indicates the number of embryos analyzed. All experiments were conducted in at least three independent cycles. All scale bars in this figure = 10 μm.

To gain insight into the possible colocalization of TDRD7 and Vasa, we analyzed the signal overlap between GFP-TDRD7 and Vasa-mCherry for each z-stack containing the spindle or perinuclear region, typically across ~20 stacks with one μm intervals, with Vasa-GFP and Vasa-mCherry as a control group (Fig. 2A–B). We set the threshold high to detect only visible (0.5 μm or larger) granules on the spindle or perinuclear. With this approach, during the M-phase, the signal overlap between GFP-TDRD7 and Vasa-mCherry was comparable to that of the control group that measured Vasa-GFP and Vasa-mCherry, while it dropped to approximately two-third during the S-phase (Fig. 2C). These results suggest that TDRD7 and Vasa may colocalize in the same granules, and they do so more significantly during the M-phase compared to the S-phase.

To test this hypothesis further, we forced TDRD7’s expression at the membrane by tagging it with membrane-mCherry (mem-TDRD7). Mem-TDRD7 formed granules as it did on the spindle, yet slightly delayed in timing, only after the 16-cell stage (Fig. 2D, arrows), while TDRD7 on the spindle appeared as granules as early as the 8-cell stage. Therefore, we performed imaging at the 16–28-cell stage for this experiment. The TDRD7 membrane granules recruited Vasa-GFP granules to the membrane (Fig. 2D, “WT”). The colocalization analysis and intensity analysis within the colocalized areas suggest that mem-TDRD7 and Vasa granules exhibit significant overlap at the membrane compared to the control group introduced with mem-mCh (Fig. 2E).

To test the specificity of Vasa recruitment, we also introduced the Vasa-C3 mutant, instead of Vasa-Wildtype (WT) (Fig. 2D, “C3”). This mutant has three amino acid mutations at the C-terminal end of Vasa (from ESWD to ASAA), which previously showed little functionality in localized translation on the spindle (Fernandez-Nicolas et al., 2022). This mutant showed significantly reduced recruitment to mem-TDRD7 (Fig. 2F). These observations suggest that mem-TDRD7 selectively recruits the WT but not the C3 mutant of Vasa. Therefore, the C3 region may be necessary for Vasa’s interaction with TDRD7.

### 3.4. The eLotus domain and the intrinsically disordered region (IDR) of TDRD7 are necessary for its interaction with Vasa

**Fig. 3.**
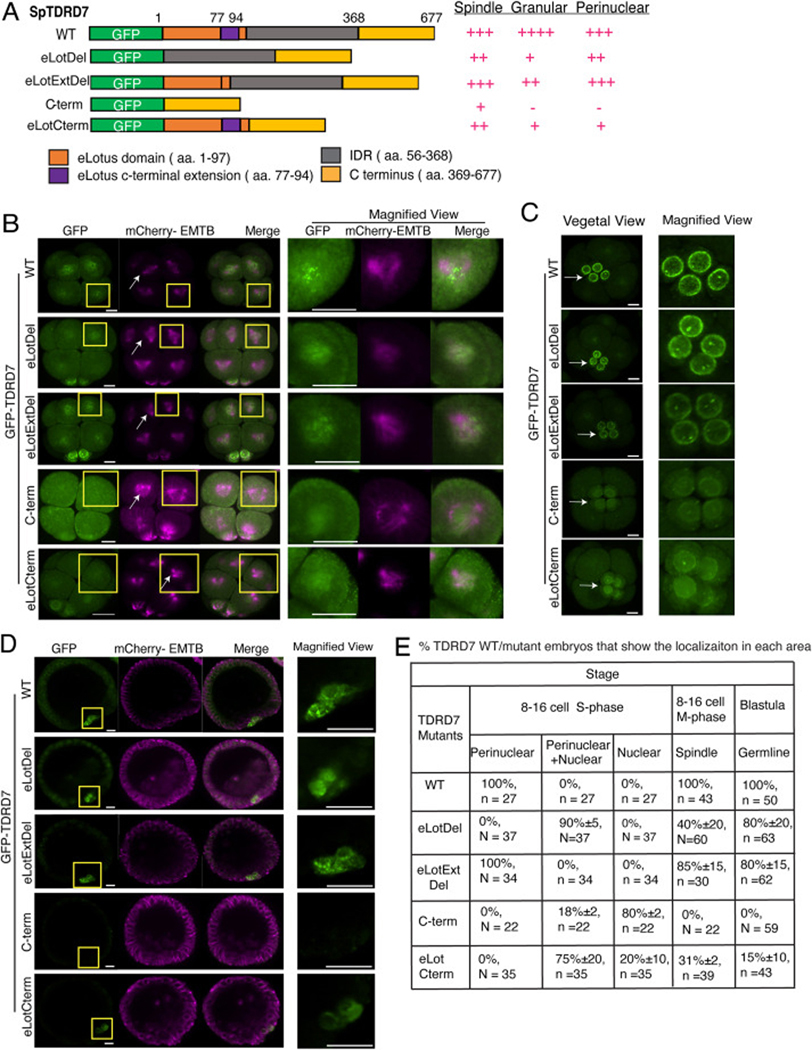
The IDR region of TDRD7 is critical for localization on the spindle and at the perinuclear region. (A) Schematic representation of TDRD7 wildtype (WT) and Mutant Constructs. (B) Representative Images of GFP-TDRD7 WT and mutants (green; 500 ng/μL stock) counterstained with microtubule marker Cherry-EMTB (magenta; 200 ng/μL stock). Squared regions are shown in the magnified views on the right. (C) Representative images of GFP-TDRD7 WT and mutants (green; 500 ng/μL stock) signals in micromeres from the vegetal view at the 16-cell stage. Arrows indicate the localization of TDRD7 in micromeres. Magnified views on the right. (D) Representative images of GFP-TDRD7 WT and mutants (green; 500 ng/μL stock) signals in the germline at the Blastula Stage. Squared regions are shown in the magnified views on the right. (E) Tabular data for the percentage of embryos with TDRD7 WT/mutant localization in each area. All experiments were conducted in at least three independent cycles. All scale bars in this figure = 10 μm.

In *Drosophila*, the eLotus domain and IDR region of Tejas/TDRD5 are reported to be necessary for its interaction with Vasa (Jeske et al., 2017; Kubíková et al., 2020; Lin et al., 2023). In the sea urchin, TDRD5 appears to be nearly undetectable during early embryogenesis, yet TDRD7 also has such a domain and a region. Therefore, we hypothesize that TDRD7 is the primary partner of Vasa in these embryonic cells instead of TDRD5. To test this hypothesis, we created GFP-tagged TDRD7 deletion mutants, each of which lacks the predicted eLotus domain and IDR region (Fig. 3A). We first visualized each mutant’s localization patterns at the 16-cell and blastula stages. mChery-EMTB was co-introduced to mark microtubules (von Dassow et al., 2009). All mutants showed reduced granule structure on the spindle at the 8–16-cell stage (Fig. 3B). Among those, the eLotExtDel mutant that lacks only the α4 region of the eLotus domain showed minimal impacts, suggesting that the α4 region is likely not responsible for TDRD7’s localization on the spindle. In contrast, the entire eLotus domain and the IDR region appear to be critical for its localization and granule formation on the spindle.

At S-phase, the WT exhibited perinuclear localization, while all mutants except for the eLotExtDel displayed ectopic signals in the nucleus (Fig. 3C). The C-term mutant, which has only the C-terminus, is primarily localized in the nucleus. In contrast, the eLotDel, which possesses the IDR and C-term domains, showed signals both at the perinuclear region and in the nucleus. The eLotCterm, which contains the eLotus domain and the C-terminus, displayed similar signal patterns to the eLotDel. These results suggest that the eLotus domain may be critical for TDRD7’s perinuclear localization, while the IDR region may be necessary for granule formation (Fig. 3C). At the late blastula stage, all mutants except for C-term showed some signal enrichment in the germline (Fig. 3D). However, the eLotDel showed the nuclear signal, unlike the perinuclear signal observed in the WT and eLotExtDel (Fig. 3D). This suggests again that the eLotus domain may be responsible for the perinuclear localization of TDRD7. In contrast, 0 % and 15 % of the C-term and the eLotCterm, respectively, showed the signal enrichment in the germline (Fig. 3D). These observations, summarized in Fig. 3E, suggest that the IDR region may be critical for the germline localization.

**Fig. 4.**
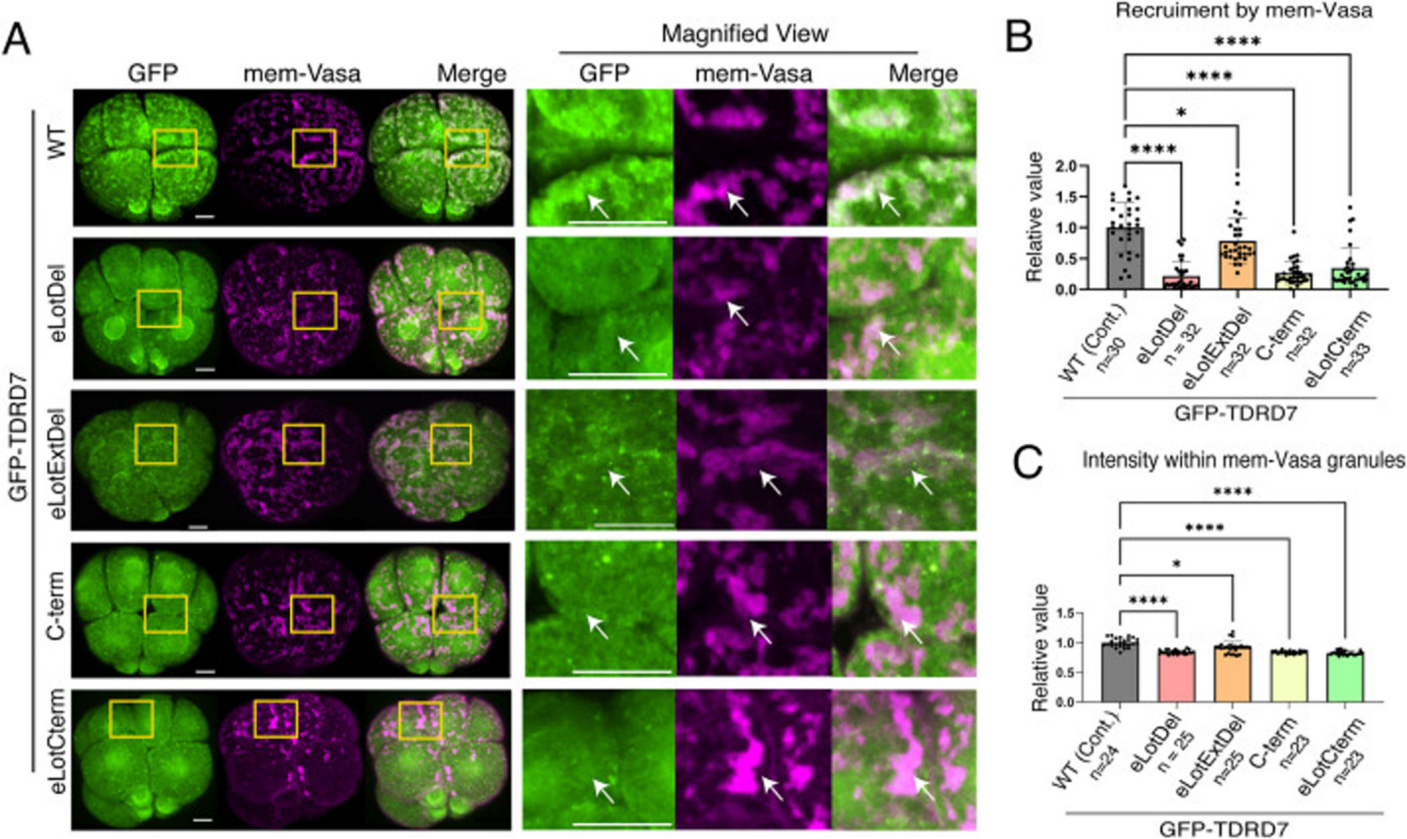
eLOTUS and IDR regions of TDRD7 are essential for interaction with Vasa. (A) Representative images of membrane-mCherry-Vasa (mem-Vasa; 1000 ng/μL stock; magenta) and GFP-TDRD7-WT/mutants (500 ng/μL stock; green). Squared regions are shown in the magnified views on the right. Arrows indicate a region enriched with Mem-Vasa granules. (B–C) Colocalization and (B) colocalization intensity (C) analysis of mem-Vasa and TDRD7-WT/mutants. Each value was normalized to the mean value of the control group in each graph. One-way ANOVA and Brown-Forsythe and Welch Anova was used for the statistical analysis. Each dot in the graph represents an individual embryo. The dots in each column of the graph indicate individual embryos. n in the graphs indicates the number of embryos analyzed. All experiments were conducted in at least three independent cycles. All scale bars in this figure = 10 μm.

To test whether these domains of TDRD7 are critical for its interaction with Vasa, we forced Vasa expression at the membrane (mem-Vasa). Similar to mem-TDRD7, mem-Vasa formed granules at the membrane and recruited TDRD7-WT (Fig. 4A–B). All of the TDRD7 mutants showed reduced recruitment to mem-Vasa. The colocalization analysis suggests that eLotExtDel is somewhat recruited to mem-Vasa; however, its granules appear much smaller and lack the lattice-like characteristics of those in the WT. The intensity analysis within the co-localized regions also yielded similar results, indicating that all mutants were recruited significantly less to the wild-type (WT) cells. Among those, however, the eLotExtDel mutant was still recruited to some extent (Fig. 4C). It remains to be determined in the future whether the eLotExtDel mutant will have a similar but lesser function to the WT in these embryonic cells.

These results again suggest that both the eLotus domain and the IDR region are essential for TDRD7’s interaction with Vasa. Furthermore, the α4 region of the eLotus domain seems critical for TDRD7’s granule characteristics, but potentially less so for its recruitment to Vasa. Overall, these results imply that the putative Vasa-binding domains of TDRD7 predicted in the germline of other organisms are also critical in embryonic cells of the sea urchin, indicating a possibility that TDRD7 and Vasa may operate similarly in the embryo as they do in the germline.

### 3.5. PiwiL proteins localize to the perinuclear and spindle regions yet are little recruited to Vasa or TDRD

We extended our exploration to Piwi proteins in this embryo since they are another group of germline factors known to work with Vasa and TDRDs in the germline. In the database, three Piwi proteins, PiwiL1 (LOC#115921666), PiwiL2 (LOC#583253), and PiwiL3 (LOC#373434) are present in the purple sea urchin. PiwiL1 and PiwiL2 have identical sequences but are encoded in different locations of the genome. Since they are indistinguishable in the mRNA sequence, we refer to them as “SpPiwiL1/2” protein in this study. Among the three Piwis, Seawi (PiwiL3) has been well characterized through past studies (Rodriguez et al., 2005; Juliano et al., 2006; Yajima et al., 2014). Previous research has shown that the PiwiL3 protein localizes to the PGCs from the blastula stage and is involved in PGC proliferation (Yajima et al., 2014). The *piwil1* mRNA expression pattern has been reported previously (Song and Wessel, 2007), yet its protein dynamics or function are yet to be known.

A phylogram analysis suggests that SpPiwiL1/2 clusters with human PiwiL2. In contrast, SpPiwiL3 does not appear to cluster with any specific human Piwi proteins (Fig. S4 A). A functional domain analysis of SpPiwis with human Piwis also indicates that SpPiwiL1/2 has the Argonaute linker1 domain, similar to human PiwiL2 (Fig. S4 B). However, this domain seems to be absent in SpPiwiL3. In the sequence alignment, the PAZ and Piwi-like domains at the C-terminus are relatively conserved across all SpPiwis and human Piwis. Therefore, the human PiwiL4 sequence was employed for the homology analysis (Fig. S4 C). The PAZ domain of SpPiwiL1/2 and SpPiwiL3 displays 50.00 % and 46.55 % homology to that of human PiwiL4, respectively. The Piwi-like domain of SpPiwiL1/2 and SpPiwiL3 shows 45.39 % and 50.51 % homology to that of human PiwiL4.

Due to the relatively conserved sequence within the PiwiL-like domain across all SpPiwis, we were able to identify the commercial human PiwiL4 antibody that may cross-react with all SpPiwi proteins (Fig. 4 C, underline). The immunoblot showed a band at the expected size of 110kDA (Fig. S5 A). Immunofluorescence showed the signal enrichment at the perinuclear, on the spindle, and in the cortex (Fig. S5 B). However, at the blastula stage, little signal enrichment was found in the germline, which is inconsistent with a previous report using the SpPiwiL3-specific antiserum (Yajima et al., 2014).

**Fig. 5.**
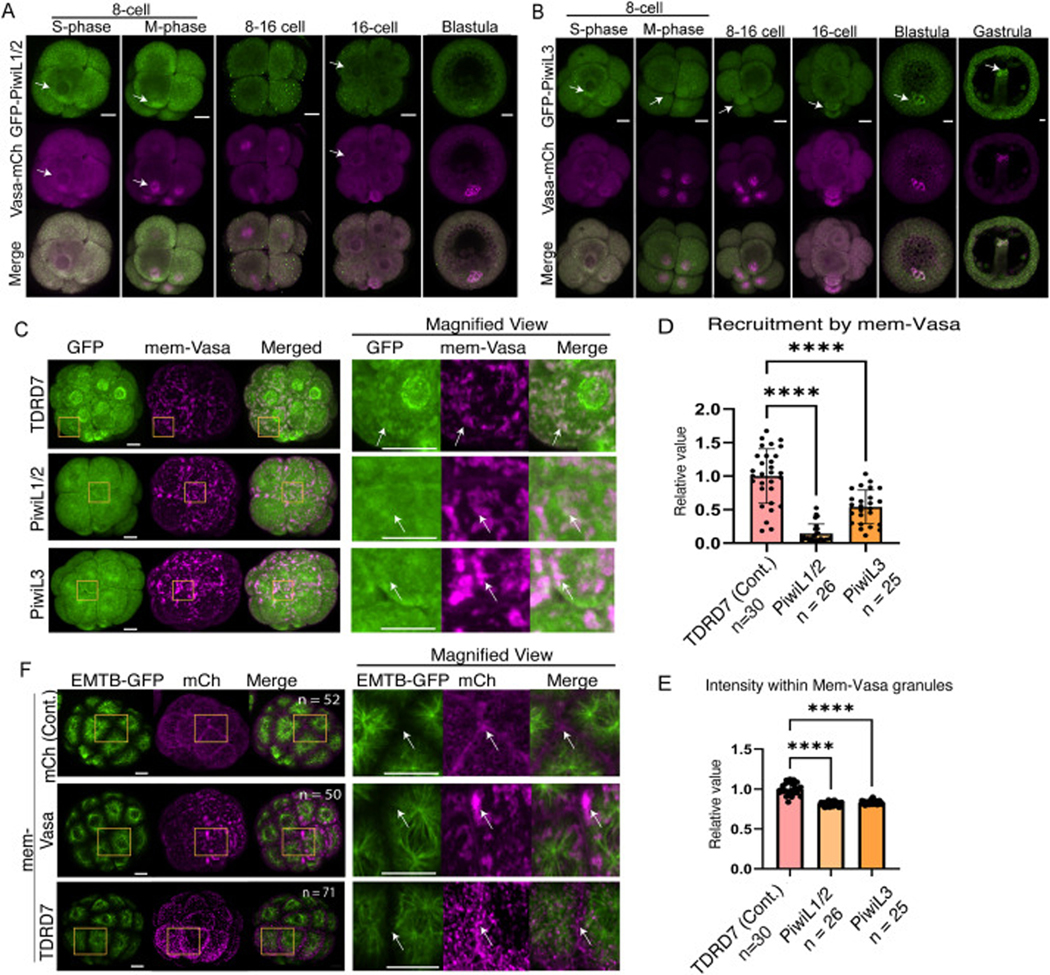
S. purpuratus PiwiL1/2 and PiwiL3 localization and interaction with Vasa. (A) Representative images of embryos expressing GFP-PiwiL1 and Vasa-mCherry at 8-cell, 16-cell, and blastula. GFP-PiwiL1/2 n = 77 (8–16 cell), n = 62 (blastula). Images obtained over six replicate, independent rounds. 75 % of S-phase embryos, 76 % of M-phase embryos, and 96 % of blastula embryos showed a similar localization signal, as shown in the image. (B) Representative images of embryos expressing GFP-PiwiL3 and Vasa-mCherry at 8-cell, 16-cell, blastula, and gastrula. Images obtained over three replicate, independent rounds. GFP-PiwiL3 n = 34 (8–16 cell), n = 24 (blastula), n = 12 (gastrula). 100 % of S-phase embryos, 76 % of M-phase embryos, 58 % of blastula embryos, and 91 % of gastrula embryos showed a similar localization signal, as shown in the image. (C) Representative images of membrane-mCherry-Vasa (mem-Vasa; 1000 ng/μL stock; magenta) and GFP-TDRD7 (Control), -PiwiL1/2, or -PiwiL3 (500 ng/μL stock; green). (D-E) Colocalization (D) and colocalization intensity € analysis of mem-Vasa and recruited TDRD7, PiwiL1/2, or PiwiL3. Each value was normalized to the mean value of the control group in each graph. The dots in each column of the graph indicate individual embryos. n in the graphs indicates the number of embryos analyzed. One-way ANOVA was used for the statistical analysis. (F) Representative images of microtubule marker EMTB-GFP (green; 100 ng/μL stock) and mem-mCh (Control), -TDRD, or -Vasa (1000 ng/μL stock; magenta). Squared regions are shown in the magnified views on the right. n in each image indicates the number of embryos analyzed. All experiments were conducted in two to four independent cycles. All scale bars in this figure = 1 0 μm.

These observations largely align with the live imaging results of GFP-SpPiwis (Piwis hereafter). GFP-PiwilL1/2 showed perinuclear localization during the S-phase and minor enrichment on the spindle during the M-phase, yet no significant signal enrichment in the germline, which Vasa-mCherry counterstains at the blastula stage (Fig. 5 A, arrows). In contrast, GFP-PiwiL3 showed a clear signal enrichment in the germline after the blastula stage (Fig. 5B, arrows), which is consistent with the previous report (Yajima et al., 2014). Therefore, the human PiwiL4 antibody used in this study appears to be preferentially detecting SpPiwiL1/2 rather than SpPiwiL3. Overall, the signal localization on the spindle and at the perinuclear region in all somatic blastomeres was similar among all Piwi proteins during early embryogenesis. The database also suggests that all *piwil* transcripts exhibit similar expression profiles during these early stages, broadly aligning with the protein dynamics (Fig. S5C).

All PiwiL protein signals were less granular than those of Vasa or TDRD7. Notably, mem-Vasa showed significantly less recruitment of PiwiL1/2 or PiwiL3 compared to TDRD7 at the 16-cell stage (Fig. 5C–D). suggesting that their interaction with Vasa may be less direct or transient. Furthermore, in *Drosophila*, Oskar, one of the TDRDs, binds to microtubules and recruits and maintains Vasa at the pole cells. Therefore, we sought to test whether mem-TDRD7 binds to the microtubules and recruits and maintains Vasa on the spindle in this embryo. For this, we analyzed the recruitment of EMTB (a microtubule marker) to mem-Vasa or mem-TDRD7. However, we did not observe any recruitment of the EMTB signal under this experimental condition (Fig. 5E). Another factor may be necessary to recruit Vasa and TDRD7 to the spindle, or a membrane-targeted approach may be insufficient to recruit the spindle, a solid structure in the cell.

## 4. Discussion

In this study, we characterized Vasa’s interacting partners in the germline and found that some of them localize on the spindle in both somatic cells and PGCs during sea urchin embryogenesis. Among those, TDRD1 plays an essential role in spermatogenesis and has been shown to localize with PiwiL2 homologs in the nuage of *Drosophila* and mouse testis (Arkov et al., 2006; Chuma, 2006; Mathioudakis et al., 2012). Similarly, TDRD7, TDRD9, and TDRD12 have been reported to localize in the nuage of mouse male germ cells (Shoji et al., 2009; Aravin et al., 2009; Voronina et al., 2011; Hosokawa et al., 2007; Pandey et al., 2013;. Furthermore, TDRD5 and TDRD7 are reported to localize in the PGCs of *Drosophila* and mouse embryos, while the localization of TDRD1, TDRD9, and TDRD12 is unclear in this context. In this study, we found that TDRD1, TDRD9, and TDRD12 showed slight enrichment on the spindle and at the perinuclear region but do not exhibit granular characteristics or enrichment in the germline during the embryogenesis of the sea urchin, whereas TDRD7 does. TDRD5, which plays a crucial role in *Drosophila* PGC, was nearly undetectable in the sea urchin embryo, at least during early embryogenesis, in this study. However, this does not exclude the possibility that TDRD5 interacts with Vasa and plays critical roles in germline development later in development. Overall, these observations are partly consistent with prior studies in *Drosophila* or mice and suggest that specific TDRDs may be designated for embryonic cell regulation.

TDRD7 showed the consistent colocalization with Vasa on the spindle of somatic lineages as well as in the germline, by forming similar granule patterns. Molecular dissection of TDRD7 suggests that the eLotus domain and the IDR region are critical for TDRD7’s granule formation and spindle localization. The mutant that lacks only the α4 of the eLotus domain exhibited relatively normal granule formation on the spindle and in the germline; however, the granules appeared much smaller and smoother when ectopically recruited to mem-Vasa at the membrane. This result suggests that the α4 region may be critical for proper granule formation of TDRD7. However, the surrounding environment could significantly influence the condensation and phase separation of molecules (Nott et al., 2015; Schmidt et al). Thus, the molecularly dense climate of the spindle likely masked a subtle change in TDRD7 granules. This also suggests that recruiting the molecules of interest to the ectopic subcellular space, such as the membrane, may provide some advantages in revealing the nature of molecules and their interactions by reducing the background. Coincidentally, the mutant lacking the α4 region also showed a reduced recruitment to mem-Vasa compared to the TDRD7-WT, suggesting that the proper granule formation of TDRD7 may be necessary for its binding to Vasa. This observation mostly aligns with the earlier findings in *Drosophila* that Oskar is responsible for organizing polar granules, which are required for recruiting Vasa to the pole plasm (Hay et al., 1990; Jones and Macdonald, 2007). The granule formation of TDRD7 may thus be necessary for its binding function to Vasa both in the germline and somatic cells.

Oskar directly interacts with microtubules, which are necessary for the recruitment and maintenance of other germline factors in PGCs of *Drosophila* (Brendza et al., 2000; Zimyanin et al., 2007). We sought to determine whether TDRD7 interacts similarly with microtubules, recruiting Vasa to the spindle in the embryo. However, mem-TDRD7 failed to recruit microtubules to the ectopic region in this study. This suggests that another factor is responsible for microtubule binding or that this ectopic recruitment approach is insufficient when the target is a large structure, such as the spindle. It is thus still unknown what recruits Vasa or TDRD to the spindle region in this embryo, and further investigation is required in the future.

In the *Drosophila* germline, it is proposed that Piwis are located near the TDRD-Vasa granule complex and transiently enter the granule complex to process piRNA biogenesis (Lin et al., 2023). Similar to this model, in this study, PiwiL1/2 and PiwiL3 exhibited spindle and perinuclear localizations, but with minimal granule formation or recruitment to TDRD7 or Vasa. It is intriguing to hypothesize that Piwis transiently enter these Vasa-TDRD granules and facilitate piRNA biogenesis on the spindle, as reported in the Bombyx mori germline (Xiol et al., 2014; Yamazaki et al., 2023; Lin et al., 2023). However, piRNA biogenesis in the germline typically occurs at the perinuclear region, and it remains to be determined whether the Vasa-TDRD7 complex has a similar role on the spindle in somatic cells. Further studies are awaited to understand how the dynamic interactions of these germline factors influence the regulation of multipotent somatic cells, such as controlling localized translation and piRNA biogenesis, during embryogenesis.

## Supplementary Material

S1-S5 supplement Figures

Tables S1-S3

## Data Availability

Data will be made available on request.

## Appendix A. Supplementary data

Supplementary data to this article can be found online at https://doi.org/10.1016/j.ydbio.2025.07.016.
