# Supplementary figures and images for: Germline factors, TDRD and Piwi, colocalize with Vasa on the mitotic apparatus during the embryogenesis of the sea urchin
Source: Dev Biol. Author manuscript; Available in PMC 2026 Feb 22. (PMC12925329; doi:10.1016/j.ydbio.2025.07.016)

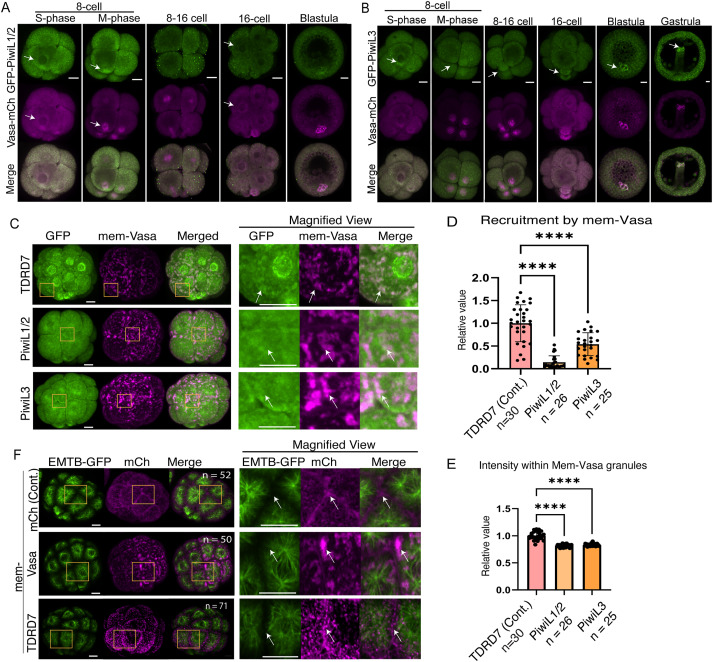

Supplement: S1-S5 supplement Figures [file NIHMS2124385-supplement-S1-S5_supplement_Figures.zip › figures/fig_05.jpg]

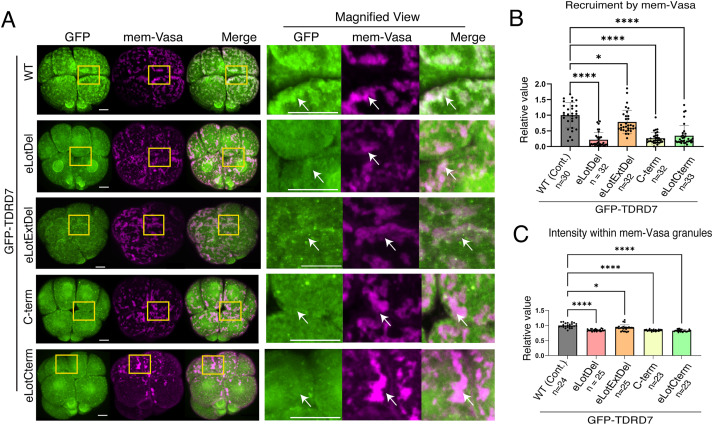

Supplement: S1-S5 supplement Figures [file NIHMS2124385-supplement-S1-S5_supplement_Figures.zip › figures/fig_04.jpg]

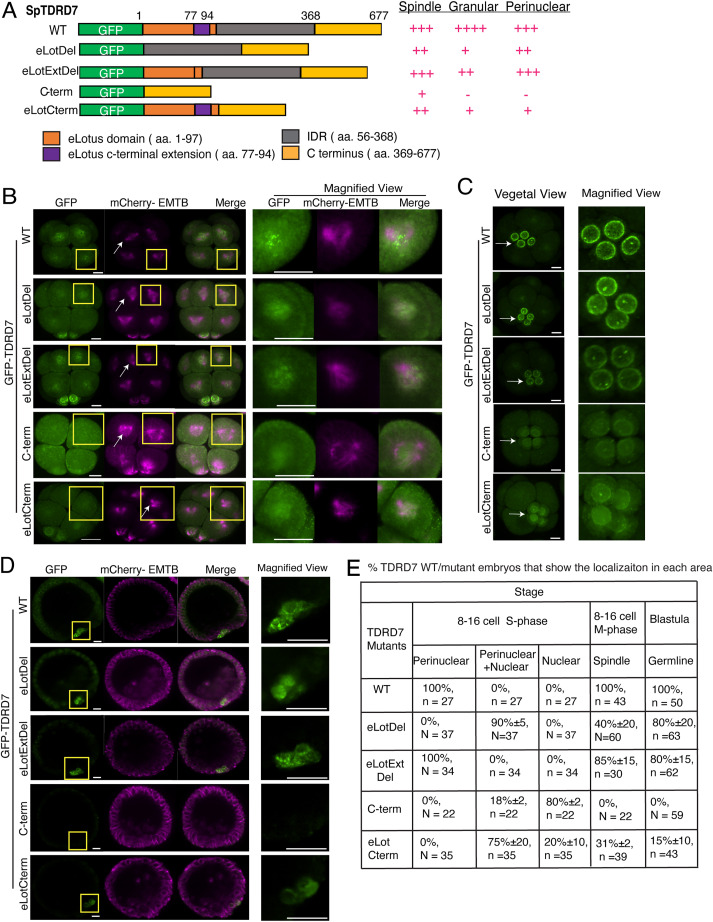

Supplement: S1-S5 supplement Figures [file NIHMS2124385-supplement-S1-S5_supplement_Figures.zip › figures/fig_03.jpg]

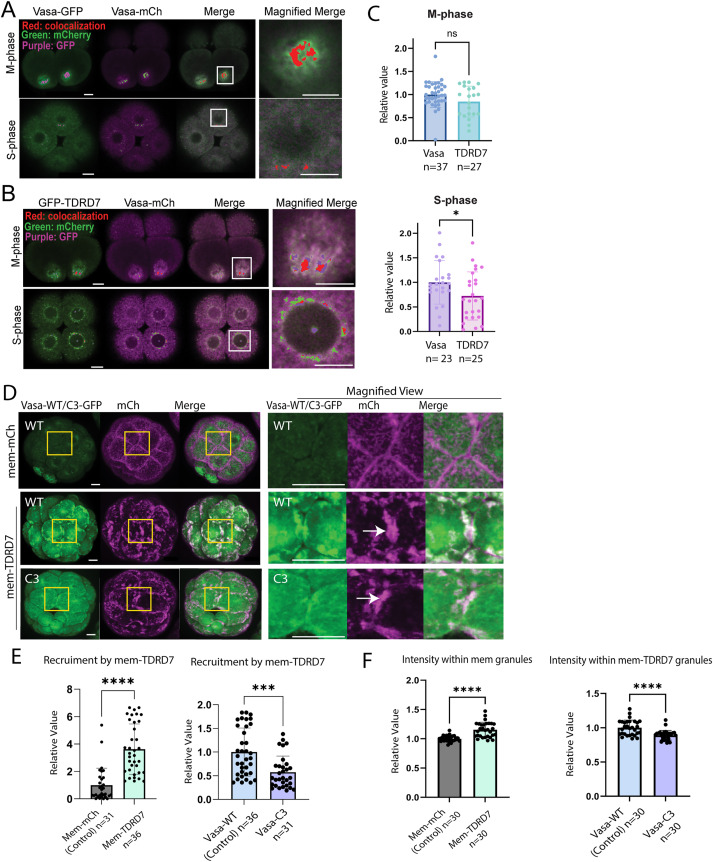

Supplement: S1-S5 supplement Figures [file NIHMS2124385-supplement-S1-S5_supplement_Figures.zip › figures/fig_02.jpg]

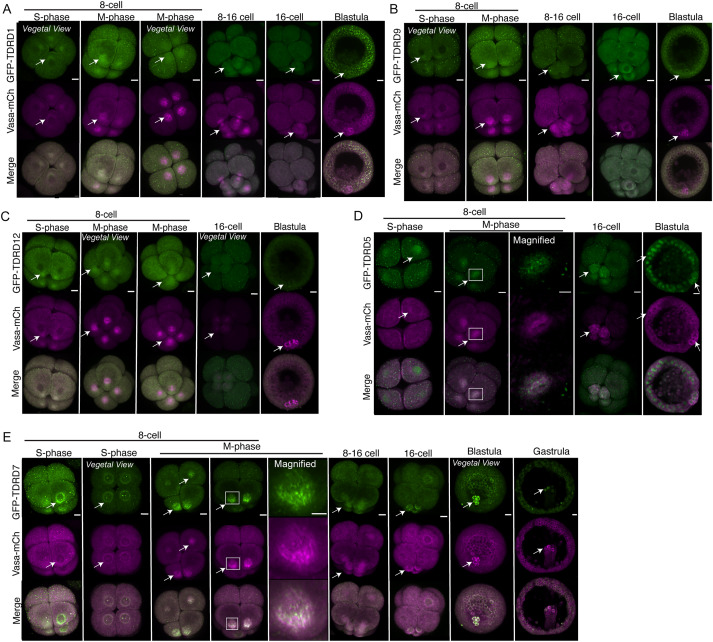

Supplement: S1-S5 supplement Figures [file NIHMS2124385-supplement-S1-S5_supplement_Figures.zip › figures/fig_01.jpg]

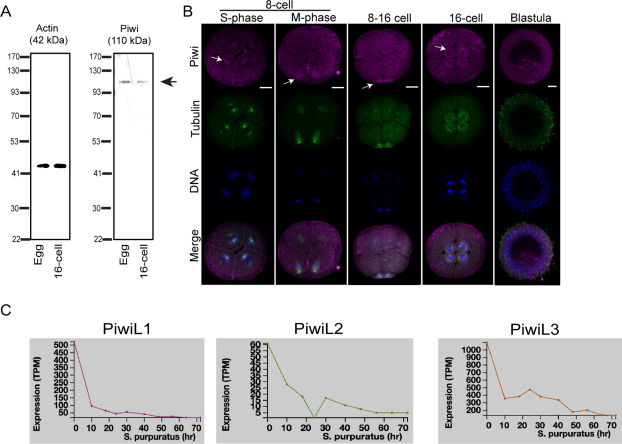

Supplement: S1-S5 supplement Figures [file NIHMS2124385-supplement-S1-S5_supplement_Figures.zip › figures/S5.jpg]

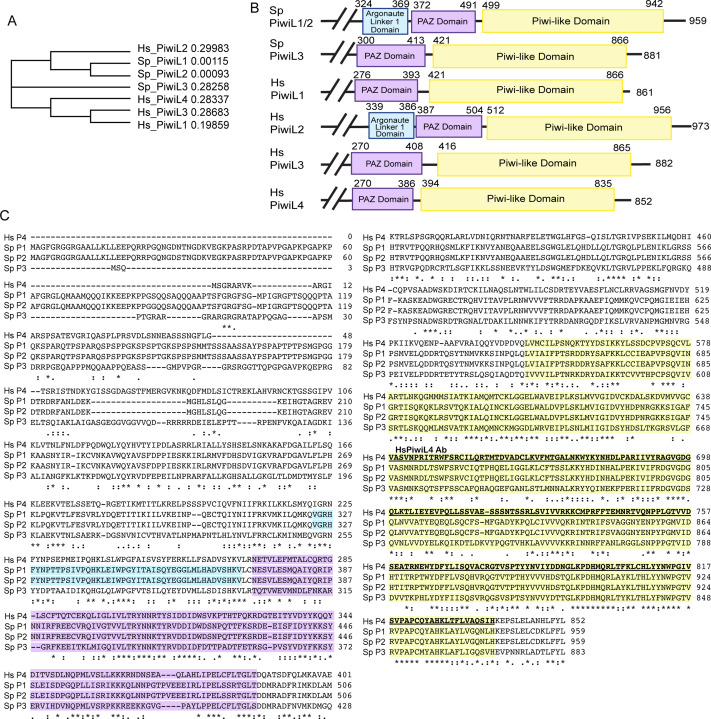

Supplement: S1-S5 supplement Figures [file NIHMS2124385-supplement-S1-S5_supplement_Figures.zip › figures/S4.jpg]

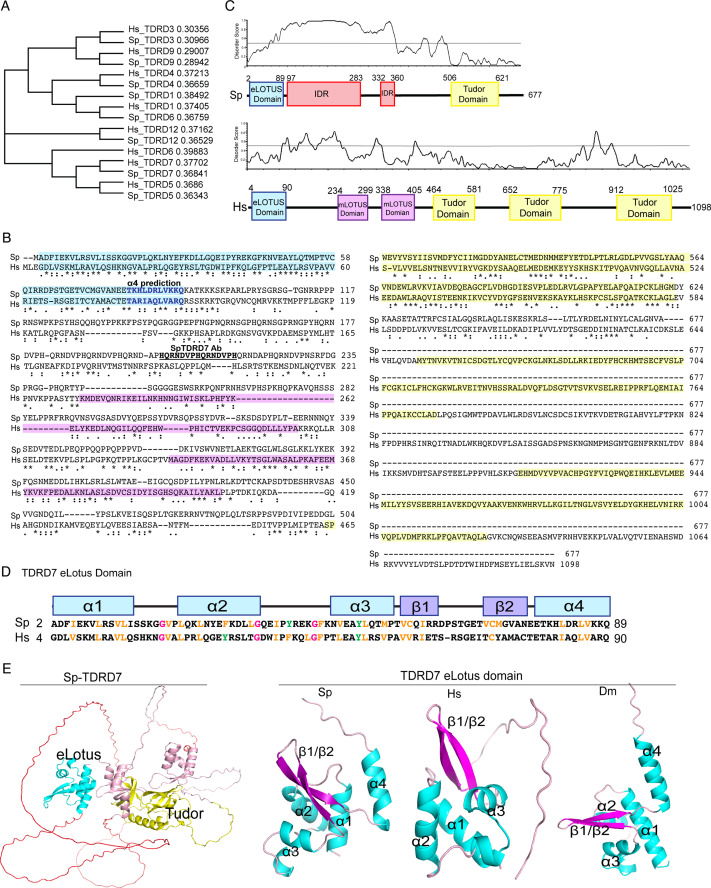

Supplement: S1-S5 supplement Figures [file NIHMS2124385-supplement-S1-S5_supplement_Figures.zip › figures/S1.jpg]

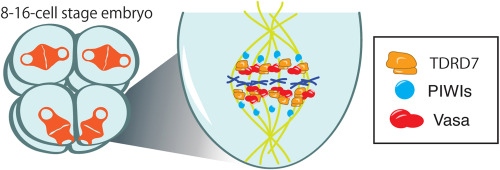

Supplement: S1-S5 supplement Figures [file NIHMS2124385-supplement-S1-S5_supplement_Figures.zip › figures/graphical-abstract.jpg]

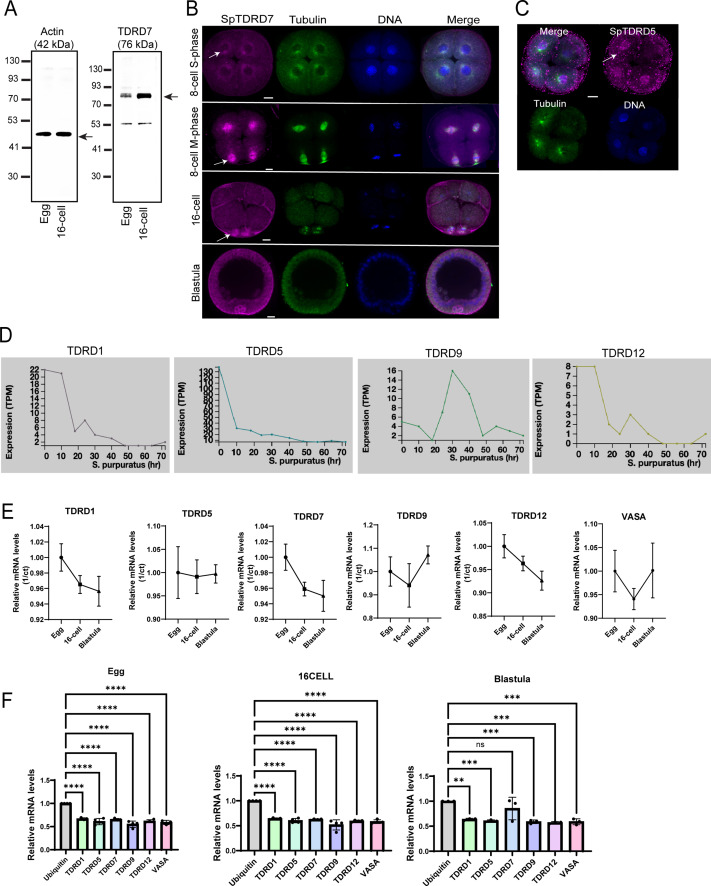

Supplement: S1-S5 supplement Figures [file NIHMS2124385-supplement-S1-S5_supplement_Figures.zip › figures/S3.jpg]

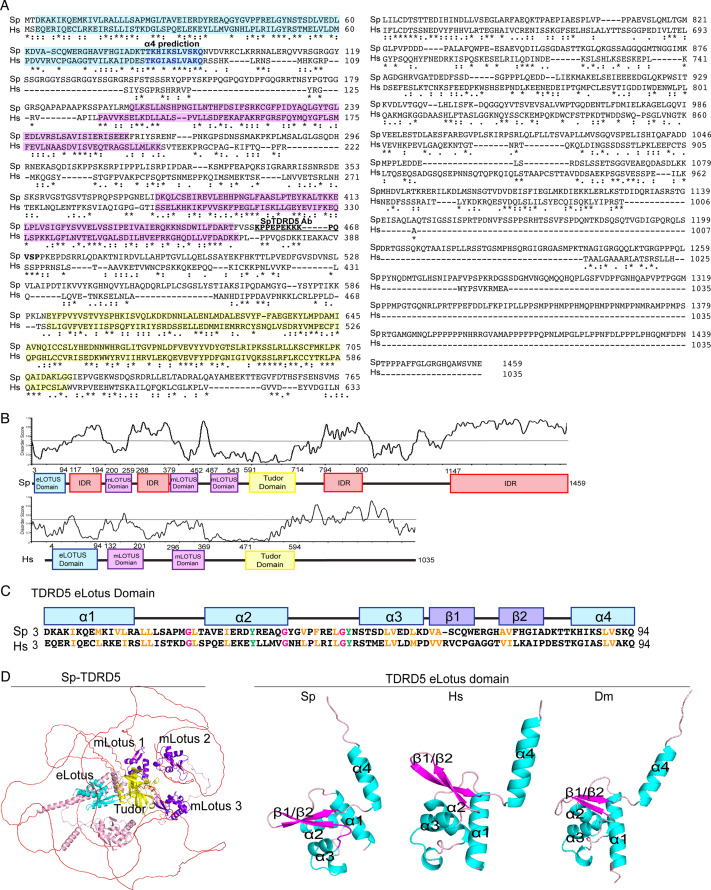

Supplement: S1-S5 supplement Figures [file NIHMS2124385-supplement-S1-S5_supplement_Figures.zip › figures/S2.jpg]
