# Supplementary material for: Germline factors, TDRD and Piwi, colocalize with Vasa on the mitotic apparatus during the embryogenesis of the sea urchin
Source: Dev Biol. Author manuscript; Available in PMC 2026 Feb 22. (PMC12925329; doi:10.1016/j.ydbio.2025.07.016)
Supplement: Tables S1-S3 [file NIHMS2124385-supplement-Tables_S1-S3.docx]

**RESOURCE TABLES**

***Table S1***

| **Protein IDs used in Subcloning, Phylogenetics Trees, and IDR Predictions** | | |
| --- | --- | --- |
| **Species**  **(Common Name)** | **Protein** | **NCBI Reference Sequence** |
| *Homo sapiens*  (Human) | DDX4 | NP_077726.1 |
|  | TDRD5 | NP_001186014.1 |
|  | TDRD7 | NP_055105.2 |
|  | PIWIL1 | NP_004755.2 |
|  | PIWIL2 | NP_001129193.1 |
|  | PIWIL3 | NP_001008496.2 |
|  | PIWIL4 | NP_689644.2 |
| *Mus musculus*  (Mouse) | DDX4 | XP_011242923 |
|  | TDRD5 | NP_001128213.1 |
|  | TDRD7 | NP_001277404.1 |
| *Drosophila melanogaster*  (Fruit Fly) | Vasa | NP_723899.1 |
|  | Tejas | NP_610950.2 |
|  | Tapas | NP_611475.3 |
| *Xenopus laevis*  (African Clawed Frog) | TDRD5 | XP_018111790.1 |
|  | TDRD7 | XP_018099138. |
| *Bombyx Mori*  (Silk Worm) | Vasa | NP_001037347.1 |
| *Strongylocentrotus Purpuratus*  *(*Purple Sea Urchin) | Vasa | NP_001139665.1 |
|  | TDRD1 | XP_030841750.1 |
|  | TDRD5 | XP_030853112.1 |
|  | TDRD7 | XP_011669388.2 |
|  | TDRD9 | XP_011664854.1 |
|  | TDRD12 | XP_030831270.1 |
|  | PIWIL1 | XP_030835319.1 |
|  | PIWIL2 | XP_030835354.1 |
|  | PIWIL3 | XP_030846820.1 |

***Table S2***

| **List of all materials used in this study** | | | |
| --- | --- | --- | --- |
| *Reagent or resource* | | *Source* | *Identifier* |
| **Antibodies** | | | |
| anti-TDRD5/TDRD7, rabbit | | This manuscript | N/A |
| anti-PIWIL4 | | ThermoFisher | #PA5-31448 |
| anti-Vasa | | Voronina et al., 2008 | N/A |
| FITC-Tubulin, mouse | | Sigma-Aldrich | #F2043 |
| Alexa 488-conjugated goat anti-rabbit IgG | | Cell Signaling Technology | # 4412 |
| Alexa 555-conjugated goat anti-mouse IgG | | Cell Signaling Technology | #4409 |
| Cy3 goat-anti-rabbit IgG | | Life Technologies | #10520 |
| Anti-β-Tubulin-FITC, mouse | | Sigma-Aldrich | #F2043 |
| **Chemicals, Peptides, and Recombinant Proteins** | | | |
| Hoechst 33342 | | Thermo Fisher Scientific | # 62249 |
| DTT | | Millipore-Sigma | # 10197777001 |
| Tris Buffered Saline, with Tween (TBST) | | Sigma-Aldrich | # 1002985083 |
| Tris-MOPS-SDS Running Buffer | | Genscript | # M00138 |
| Transfer Buffer Powder | | Genscript | # M00139 |
| Phosphate Buffered Saline | | Sigma-Aldrich | # 1002957006 |
| **Critical Commercial Assays** | | | |
| mMESSAGE mMACHINE SP6 Transcription Kit | | Ambion | # AM1340 |
| In-Fusion HD Cloning | | Clontech | # 639648 |
| RNeasy Plus Mini Kit | | Qiagen | #74034 |
| PrimeScript™ RT Master Mix (Perfect Real Time) | | Takara | #RR036A |
| Luna qPCR Universal Master Mix | | New England BioLab | # M3003X |
| **Recombinant DNA** | | | |
| Plasmid: 2xmCherry-EMTB | | Addgene | # 26742 |
| **Cloning Primers** | | | |
| Sp-TDRD1 | | F1: ATGGATTTTTATCGATTATTGAG  F2: GGAGACAGCTGAGCAGAAGAAGGC  R1: GCAGTGCTTCGAACCTGTGGGGG  R2: TCAGGTGGACATTCCCCTGGCC | |
| Sp-TDRD5 | | F1: ATGACAGACAAGGCCAAAATCAAGC  F2: CTGTTCCCTCTACCATGAAG  R1: GGAACACCTGTAATGAGGCCCC  R2: TTACTCATTGACCGACCATGCTTGG | |
| Sp-TDRD7 | | F1: ATGGCCGACTTTATCGAGAAAG  F2: GGTCCAAGTCAGACAGTGACTACCC  R1: GGGGTGGTTGTTGAGGGGGCTGCTC  R2: CTAGGCCACATTTCCTAGGGCAC | |
| Sp-TDRD9 | | F1: ATGGCTAGATTTCTAACTGCAGAAG  F2: GGAGCTGGTCTGTCTCTCAAAAGC  R1: GGCAACAGGATCGCTGAAGCTTCC  R2: TCACACTAAAGGAGGGGGGTCC | |
| Sp-TDRD12 | | F1: ATGATGACCAACATCACCATTTTA  F2: GAGCTCAACTCAGCCATCCCTTTC  R1: CAAGAGGTTTGTGGATCCCTGG  R2: TTAGAAATAATAGCTCACCTC | |
| Sp-PIWIL1 | | F1: ATGGCAGGCTTCGGACGCGGAGGG  F2: GGACAGCCGTTACTCATCAG  R1: CTTCTTCCACTGGGGTCCCTG  R2: TCACAGGAAGAAGAGCTTGTCGC | |
| Sp-PIWIL2 | | F1: ATGGCAGGCTTCGGACGCGG  R1: GATGAGTAGTGGCTGGCCTGGGTC  R2: TCACAGGAAGAAGAGCTTGTCG | |
| Sp-PIWIL3 | | F1: ATGTCGCAACCAACAGGCAGAGCC  F2: GAGACCCGTTCCGGACCAGTATC  R1: CCAACTCCCTTCTTTTCCTCCC  R2: CTAGAGATAGAAGAGGGTGTCGGC | |
| **Infusion Primers** | | | |
| Sp-TDRD1 | F: CATGGACGAGCTGTACAAGTGCGGCCGCATGGATTTTTATCGATTATTGAG  R1: CCAGATCCTAGTCAGTCACTAGTGCAGTGCTTCGAACCTGTGGGGG  R2:  CCAGATCCTAGTCAGTCACTAGTCAGGTGGACATTCCCCTGGCC | | |
| Sp-TDRD5 | F: CATGGACGAGCTGTACAAGTGCGGCCGCATGACAGACAAGGCCAAAATCAAGC  R1: CCAGATCCTAGTCAGTCACTAGTGGAACACCTGTAATGAGGCCCC  R2: CCAGATCCTAGTCAGTCACTAGTTTACTCATTGACCGACCATGCTTGG | | |
| Sp-TDRD7 | F: CATGGACGAGCTGTACAAGTGCGGCCGCATGGCCGACTTTATCGAGAAAG  R: CCAGATCCTAGTCAGTCACTAGTCTAGGCCACATTTCCTAGGGCAC | | |
| Sp-TDRD9 | F: CATGGACGAGCTGTACAAGTGCGGCCGCATGGCTAGATTTCTAACTGCAGAAG  R1: CCAGATCCTAGTCAGTCACTAGTGGCAACAGGATCGCTGAAGCTTCC  R2: CCAGATCCTAGTCAGTCACTAGTTCACACTAAAGGAGGGGGGTCC | | |
| Sp-TDRD12 | F: CATGGACGAGCTGTACAAGTGCGGCCGCATGATGACCAACATCACCATTTTA  R1: CCAGATCCTAGTCAGTCACTAGTCAAGAGGTTTGTGGATCCCTGG  R2: CCAGATCCTAGTCAGTCACTAGTTAGAAATAATAGCTCACCTC | | |
| Sp-PIWIL1 | F: CATGGACGAGCTGTACAAGTGCGGCCGCATGGCAGGCTTCGGACGCGG  R: CCAGATCCTAGTCAGTCACTAGTTCACAGGAAGAAGAGCTTGTCG | | |
| Sp-PIWIL2 | F: CATGGACGAGCTGTACAAGTGCGGCCGCATGGCAGGCTTCGGACGCGG  R: CCAGATCCTAGTCAGTCACTAGTTCACAGGAAGAAGAGCTTGTCG | | |
| Sp-PIWIL3 | F: CATGGACGAGCTGTACAAGTGCGGCCGCATGTCGCAACCAACAGGCAGAGCC  R: CCAGATCCTAGTCAGTCACTAGTCTAGAGATAGAAGAGGGTGTCGGC | | |
| **Infusion Primers for TDRD7 mutants** | | | |
| TDRD7-eLotDel | F: CATGGACGAGCTGTACAAGTGCGGCCGCCCTGCACGACTGCCGAGATATTC  R: CCAGATCCTAGTCAGTCACTAGTCTAGGCCACATTTCCTAGGGCAC | | |
| TDRD7  c-Term | F: CATGGACGAGCTGTACAAGTGCGGCCGCGTCAATGAGACGTTGGCTGAAAAG  R: CCAGATCCTAGTCAGTCACTAGTCTAGGCCACATTTCCTAGGGCAC | | |
| TDRD7  eLotcTerm | F: CATGGACGAGCTGTACAAGTGCGGCCGCATGGCCGACTTTATCGAGAAAG R: CCAGATCCTAGTCAGTCACTAGTCTAGGCCACATTTCCTAGGGCAC | | |
| Infusion Primers for Membrane TDRD7/Vasa Constructs | | | |
| Membrane-mCherry TDRD7 | F: GAGGGGATCGGTGGAGCTCCACCGGTATGGCCGACTTTATCGAGAA R: CCAGATCCTAGTCAGTCACTAGTCTAGGCCACATTTCCTAGGGCAC | | |
| Membrane-mCherry  Vasa | F: GAGGGGATCGGTGGAGCTCCACCGGTATGTCAGAAGACTGGGGGAC R: GTAACCAGATCCTAGTCAGTCACTAGTTTAATCCCATGATTCATCATCAG | | |
| **gBlock for TDRD7 mutants** | | | |
| TDRD7 eLotExtDel | CATGGACGAGCTGTACAAGTGCGGCCGCATGGCCGACTTTATCGAGAAAGTTCTTAGATCAGTCCTTATATCCAGCAAAGGTGGAGTACCTCTACAGAAACTCAACTATGAGTTCAAAGATCTCCTGGGTCAAGAGATCCCATACAGGGAAAAGGGTTTCAAGAATGTGGAGGCCTATCTGCAGACCATGCCTACAGTCTGCCAAATAAGGAGAGATCCATCTACTGGTGAGACAGTTTGTATGGGCGTTGCAAATAAGAGCAAACCTGCACGACTGCCGAGATA | | |
| **qPCR Primers** | | | |
| Sp-TDRD1 | F: TGTCACCTACGTGTGGCTTG R: CCGTCACTCAGGGATGGATG | | |
| Sp-TDRD5 | F: CACCTCCTTCCAGACACACC  R: TTTTGACTCGACCCAGACCG | | |
| Sp-TDRD7 | F: CGTTGGCTGAAAAGACTGGC R: AACACCAATCTCCTCCGCTG | | |
| Sp-TDRD9 | F: CCCCCGTAATCAGCAGAAAC R: TGGGTCTTTCCCTGACAGTATC | | |
| Sp-TDRD12 | F: GATCAGAGACGGTCAGCTCC R: TGTGAGTAATGACGACGAGC | | |
| Sp-Vasa | F: TCAACTACGACCTCCCAAGC R: TCTCGCAATGTTAGCATCCTT | | |
| **Software and Algorithms** | | | |
| Echinoderm gene/protein sequences | | EchinoBase | http://www.echinobase.org/Echinobase/ |
| Imaging Software | | Nikon NIS Elements |  |
| Statistical Analysis | | GraphPad PRISM | https://www.graphpad.com/scientific-software/prism/ |
| Phylogenetic Tree Analysis and Protein Alignment | | Clustal Omega | https://www.ebi.ac.uk/Tools/msa/clustalo/ |
| IDR Prediction | | IUPRED3 | https://iupred3.elte.hu/ |
| Conserved Domain Prediction | | NCBI Protein Blast | https://blast.ncbi.nlm.nih.gov/Blast.cgi |

***Table S3***

| **Restriction enzymes (RE) used for InFusion subcloning and In vitro transcription (IVT)** | | |
| --- | --- | --- |
| **Construct** | **Infusion RE or citation** | **IVT RE** |
| SP64-GFP-TDRD1 | SpeI | XbaI |
| SP64-GFP-TDRD5 | SpeI | SalI |
| SP64-GFP-TDRD9 | SpeI | SalI |
| SP64-GFP-TDRD12 | SpeI | SalI |
| SP64-GFP-PIWIL1 | SpeI | BamHI |
| SP64-GFP-PIWIL2 | SpeI | BamHI |
| SP64-GFP-PIWIL3 | SpeI | SalI |
| SP64-Vasa-GFP | Fernandez-Nicholas et al., 2022 | SalI |
| SP64-Vasa-mCherry | Fernandez-Nicholas et al., 2022 | SalI |
| pC52-2xmCherry-EMTB | von Dassow et al., 2009 | Not1 |
| 3xFlag-GFP-TDRD7 | ApaI | BamHI |
